# A Case of Drug Reaction With Eosinophilia and Systemic Symptoms (DRESS) Syndrome in a Patient Receiving Peritoneal Dialysis With Icodextrin Exposure

**DOI:** 10.7759/cureus.30797

**Published:** 2022-10-28

**Authors:** Manal E Alotaibi, Samantha Saggese, Ibrahim Tawhari, Lida Zheng, Cuong V Nguyen, Vikram Aggarwal

**Affiliations:** 1 Department of Medicine, Division of Nephrology, Johns Hopkins University School of Medicine, Baltimore, USA; 2 Department of Medicine, Division of Nephrology, Northwestern University Feinberg School of Medicine, Chicago, USA; 3 Department of Internal Medicine, Division of Nephrology, King Khalid University, Abha, SAU; 4 Department of Medicine, Division of Dermatology, Northwestern University Feinberg School of Medicine, Chicago, USA

**Keywords:** peritoneal dialysis (pd), skin rash, end stage renal disease (esrd), icodextrin, dress syndrome

## Abstract

Icodextrin solutions are associated with rashes within a few weeks of initial exposure. However, severe skin reactions are rarely reported. Cessation of icodextrin is necessary for treatment, though systemic steroids were used in a few cases. Drug reaction with eosinophilia and systemic symptoms (DRESS) syndrome is a severe drug reaction characterized by an extensive rash associated with eosinophilia, visceral organ involvement, lymphadenopathy, or atypical lymphocytosis. Recurrence can develop weeks to months after drug cessation, even without re-exposure. To our knowledge, DRESS has not been reported with icodextrin use. Herein, we report a case of relapsing generalized maculopapular skin rash that developed with icodextrin use, highly suggestive of DRESS syndrome.

## Introduction

Icodextrin is a starch-derived, high-molecular-weight (16,200 Da) glucose polymer. Due to its colloid osmotic property, it is used in the form of aqueous peritoneal dialysis (PD) solution that promotes better ultrafiltration (UF), compared to dextrose-based solutions during long dwell times. It is often used in patients with high average transport characteristics [1]. Icodextrin is associated with mild skin rash and pruritis that typically starts within a few days or weeks of initial exposure [1,2]. However, severe hypersensitivity skin reactions with widespread exfoliative dermatitis are rarely reported [1,2]. Drug reaction with eosinophilia and systemic symptoms (DRESS) syndrome is a severe adverse drug reaction characterized by an extensive skin rash associated with visceral organ involvement, lymphadenopathy, eosinophilia, and atypical lymphocytosis. DRESS syndrome has not been reported with icodextrin use.

We report a challenging case of relapsing generalized maculopapular skin rash that developed with an icodextrin-based PD solution. The patient also had peripheral eosinophilia and diagnostic features of DRESS syndrome.

## Case presentation

A 77-year-old woman with chronic kidney disease stage 5 due to biopsy-proven diabetic nephropathy opted to initiate PD to manage worsening fluid overload. She received one dose of cefazolin as preoperative prophylaxis. PD catheter placement and initial catheter flushes were uneventful. Three weeks after catheter placement, her daughters started continuous ambulatory peritoneal dialysis (CAPD) training in our home dialysis center. After one week, an icodextrin-based solution was used instead of a dextrose-based solution to avoid hyperglycemia and achieve better UF. After two weeks of a successful regimen of two icodextrin exchanges per day, the patient was discharged to initiate PD at home. A day after discharge, she developed a mild pruritic skin rash on her abdomen. Moisturizing cream was prescribed, and within one week, she presented to the hospital with a worsening pruritic skin rash that had migrated from her abdomen to her chest, arms, and back. There were no recent medications change or known allergies. Her home medications included amlodipine, sodium bicarbonate, and insulin glargine. Her vital signs were stable.

**Figure 1:**
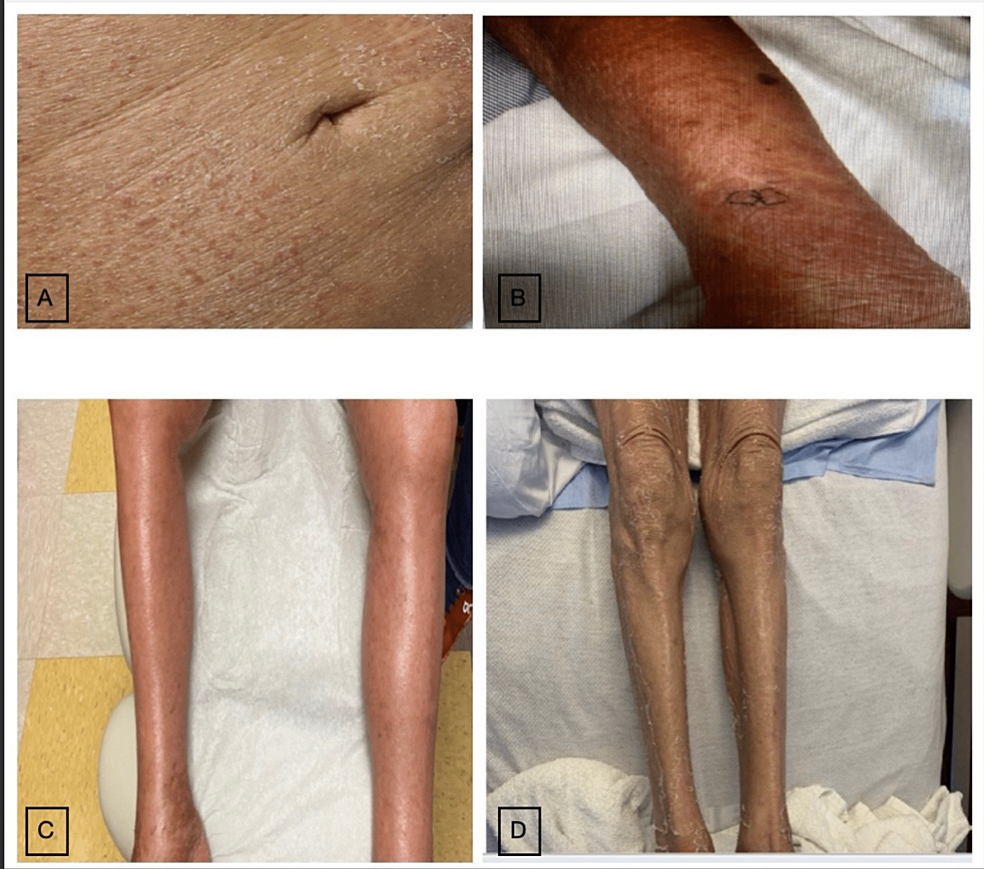
A: Erythematous, maculopapular skin rash expanding abdomen, B: worsening skin rash on the upper extremities, C: Diffuse erythema on the lower extremities, and D: Diffuse exfoliative dermatitis expanding on the lower extremities.

**Figure 2:**
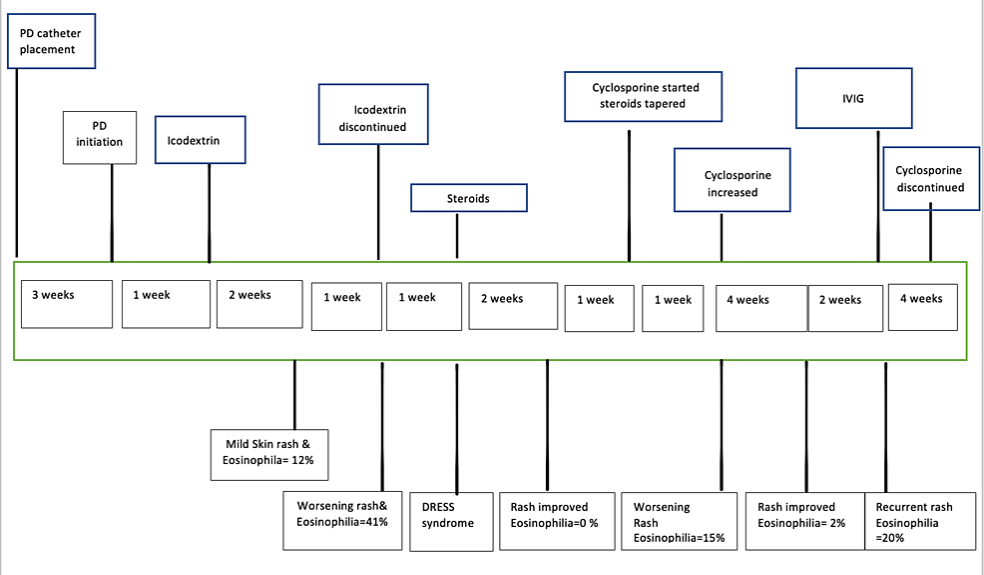
Timeline of skin rash and management PD: peritoneal dialysis; DRESS: drug reaction with eosinophilia and systemic symptoms; IVIG: intravenous immunoglobulin

The examination was notable for diffuse, erythematous, maculopapular skin rash over her chest, arms, abdomen, and back. No oral lesions or mucosal lesions were identified (Figure 1A). She did not have abdominal tenderness and the PD catheter exit site was normal. Laboratory tests revealed leukocytosis with eosinophilia (12%) and PD-associated peritonitis (Table 1). Empiric intraperitoneal (IP) vancomycin and ceftazidime were started. PD fluid culture grew E. coli, and antibiotics were narrowed to IP ceftazidime. Icodextrin was discontinued and CAPD was continued with a dextrose-based PD solution. Dermatology was consulted, and the rash was thought to be due to allergic contact or irritant dermatitis. She was prescribed triamcinolone ointment. Peripheral eosinophil counts rose throughout the admission and peaked at 41%. PD-associated peritonitis showed improvement within four days, and the skin rash was unchanged. Ten days after discharge, she was seen in the dermatology clinic for worsening skin rash with face and scalp involvement (Figure 1B). Dermatology suspected icodextrin-associated DRESS due to generalized exfoliative rash (> 50 % of body surface area), facial involvement, and persistent severe eosinophilia (absolute eosinophil count of 4000 x 109L). A skin biopsy was performed in the clinic, and she was hospitalized for close monitoring as DRESS is associated with a high mortality rate (up to 10%) [3,4]. An infectious work-up that included HTLV IgG, HHV6, COVID PCR, respiratory viral panel, mycoplasma, chlamydia, and hepatitis B and C antibodies were negative. High-dose glucocorticoid (1mg/kg) was initiated. Skin biopsy demonstrated mild interface dermatitis with eosinophilia, suggesting a drug hypersensitivity reaction. Per the scoring system for the diagnosis of DRESS syndrome, her score was 5, which was highly probable for the diagnosis of DRESS with a score of >/= 6 being definitive [3]. During this admission, she had Pseudomonas peritonitis, and she was started on IP ceftazidime; and the steroid dose was reduced. Skin rash improved, and peripheral eosinophil count normalized. During the clinic visit in the next week, she experienced active generalized skin rash and hyperglycemia. Hence, oral cyclosporine was started as a second-line therapy because of intolerance to steroids. The steroid dose was tapered. Unfortunately, after a week, she was hospitalized with severe Candida albicans peritonitis and started fluconazole with an adjustment of the cyclosporine dose. PD catheter was removed, and she transitioned to intermittent hemodialysis (iHD). She noticed a worsening skin rash during this hospitalization, with new erythema over her thighs and eosinophils of 15% (Figure 1C). The cyclosporine dose was increased, and prednisone was tapered off. Over the next four weeks, rash and eosinophilia improved dramatically, and she continued cyclosporine. She presented again with diffuse exfoliative dermatitis with seborrheic dermatitis-like lichenification on her scalp and face, and the eosinophil count rose to 20% (Figure 1D). Intravenous immunoglobulin (IVIG; 1 g/kg) was administered. Cyclosporine was discontinued as she had recurrent abdominal infections. DRESS features did not recur after this episode, skin lesions entirely healed over one month, and she remains in remission during the subsequent nine months of follow-up to date. She continues to be on iHD. Figure 2 summarized her clinical course.

**Table 1:** Summary of the laboratory studies in the first hospital admission

Measure	Reference	Result
Sodium (mmol/l)	137-145	136
Potassium (mmol/l)	3.5-5.1	2.5
Chloride (mmol/l)	98-109	95
Carbon dioxide (mmol/l)	21-31	31
Blood urea nitrogen (mg/dl)	2-25	50
Creatinine (mg/dl)	0.6-1.30	5.58
Calcium (mg/dl)	8.3-10.5	8.4
Albumin (g/dl)	3.5-5.7	2.9
White blood cell count (10ˆ3/µL)	3.5-10.5	15
Neutrophils (%)	34-73	94
Lymphocyte (%)	15-50	6
Eosinophils (%)	0.0-8	12
Absolute eosinophils (10ˆ3/µL)	0.0-0.6	1.7
Peritoneal fluid analysis		
Color	Yellow	
WBC count (/UL)	3194	
Neutrophils (%)	94	
Eosinophils (%)	2	

## Discussion

This case demonstrates an association between the rare but life-threatening DRESS syndrome and icodextrin use in a patient on PD. The literature reports an incidence of skin rash with icodextrin use as high as 18.95% [1]. However, a 2013 meta-analysis of 11 randomized clinical trials comparing icodextrin and dextrose-based solutions showed no increase in adverse events, including skin rash with icodextrin [2]. Allergic skin rashes due to icodextrin were generally maculopapular rash that started within a few days or weeks of initial exposure and was limited to the abdomen [1,2]. Our patient was initially on an icodextrin solution once daily for one week as approved by FDA [5,6]. The onset of skin rash was noticed two weeks after increasing the frequency of icodextrin exchanges. The increase in exposure to icodextrin could have been the trigger for the skin rash. However, previous studies have shown that twice-daily icodextrin is not associated with a higher incidence of skin rash [5,6]. While the mechanism of the allergic phenomenon with icodextrin remains unclear, icodextrin’s chemical structure is similar to that of dextran, which has a high incidence of allergic reactions and anaphylaxis [7,8]. Cessation of icodextrin was the only necessary intervention to resolve skin rash; however, systemic steroids were used in a few severe cases [9,10]. Our patient presented with a widespread exfoliative skin rash and marked eosinophilia that did not resolve after stopping icodextrin. Dermatologists contemplated a high probability for diagnosis of DRESS and strongly suspected icodextrin as the causative agent. DRESS syndrome has not previously been reported with icodextrin use. DRESS syndrome is a life-threatening hypersensitivity reaction associated with fever, lymphadenopathy, hepatitis, hematologic abnormalities, eosinophilia, and atypical lymphocytes [11]. It can involve internal organs, such as the kidneys, heart, lungs, and pancreas [11]. Culprit drugs can trigger the reactivation of Herpes virus or induce a T-cell response leading to the pathogenesis of DRESS in individuals with genetic susceptibility [12]. Infectious etiologies were ruled out in her case. The course of DRESS syndrome can be prolonged, with flares after cessation of the potential cause, as in this case [12]. Recurrence can develop weeks to months after drug cessation, even without re-exposure to the potential cause [12]. Using dextrose-based solution dialysate has rarely been associated with self-limited skin rash [2]. Therefore, it is less likely that the dextrose-based solution would be the potential cause. Patients with DRESS syndrome typically respond to high-dose glucocorticoids [9], Our patient responded initially to high-dose steroids, but she had a flare of DRESS with rapid steroid taper. Cyclosporine is often used to treat DRESS syndrome when the response to steroids is poor or when their use is contraindicated, as in this case [13]. She initially responded to cyclosporine but then had a severe protracted and unremitting course. The use of immunosuppressive medications to treat DRESS syndrome was challenging in the context of recurrent PD-associated peritonitis. It is unclear if this provoked a relapse of the disease. IVIG has been successfully used in severe cases of DRESS syndrome. She eventually received IVIG with great effect.

## Conclusions

This case report highlights an association between DRESS syndrome and icodextrin use for the first time. Nephrologists should be aware of rare and life-threatening icodextrin-related DRESS while managing severe skin rash in PD patients. These challenging situations require dermatology consultations, and close monitoring and may need specific treatments beyond icodextrin cessation.
